# Cellular diversity through space and time: adding new dimensions to GBM therapeutic development

**DOI:** 10.3389/fgene.2024.1356611

**Published:** 2024-05-07

**Authors:** Amanda L. Johnson, Hernando Lopez-Bertoni

**Affiliations:** ^1^ Hugo W. Moser Research Institute at Kennedy Krieger, Baltimore, MD, United States; ^2^ Department of Neurology, Baltimore, MD, United States; ^3^ Oncology, Baltimore, MD, United States; ^4^ Sidney Kimmel Comprehensive Cancer Center at Johns Hopkins University School of Medicine, Baltimore, MD, United States

**Keywords:** glioblastoma, single-cell sequencing, spatial omics, heterogeneity, cellular plasticity, cancer therapeutics, immunotherapy, hypoxia

## Abstract

The current median survival for glioblastoma (GBM) patients is only about 16 months, with many patients succumbing to the disease in just a matter of months, making it the most common and aggressive primary brain cancer in adults. This poor outcome is, in part, due to the lack of new treatment options with only one FDA-approved treatment in the last decade. Advances in sequencing techniques and transcriptomic analyses have revealed a vast degree of heterogeneity in GBM, from inter-patient diversity to intra-tumoral cellular variability. These cutting-edge approaches are providing new molecular insights highlighting a critical role for the tumor microenvironment (TME) as a driver of cellular plasticity and phenotypic heterogeneity. With this expanded molecular toolbox, the influence of TME factors, including endogenous (e.g., oxygen and nutrient availability and interactions with non-malignant cells) and iatrogenically induced (e.g., post-therapeutic intervention) stimuli, on tumor cell states can be explored to a greater depth. There exists a critical need for interrogating the temporal and spatial aspects of patient tumors at a high, cell-level resolution to identify therapeutically targetable states, interactions and mechanisms. In this review, we discuss advancements in our understanding of spatiotemporal diversity in GBM with an emphasis on the influence of hypoxia and immune cell interactions on tumor cell heterogeneity. Additionally, we describe specific high-resolution spatially resolved methodologies and their potential to expand the impact of pre-clinical GBM studies. Finally, we highlight clinical attempts at targeting hypoxia- and immune-related mechanisms of malignancy and the potential therapeutic opportunities afforded by single-cell and spatial exploration of GBM patient specimens.

## Introduction

Despite advancement in the field of cancer therapeutics, attempts at treating patients with glioblastoma (GBM), the most common adult brain cancer with a universally fatal prognosis (Louis et al., 2021; Ostrom et al., 2023), have had limited success. GBM inevitably recurs, for which there is currently no effective treatment, and no new drugs have been FDA-approved to treat GBM since 2009 (Wen et al., 2020). However, as drug after drug fails to have significant clinical efficacy for GBM patients, our understanding of the cellular and molecular drivers of GBM and treatment resistance grows. Two major culprits identified in therapeutic inefficacy are the molecular heterogeneity and phenotypic plasticity of GBM cells (Sottoriva et al., 2013; Liau et al., 2017; Yabo et al., 2022). These cellular traits cooperate to support the spectrum of cellular states found within GBM. The heterogeneity of GBM encompasses the genome, epigenome, and transcriptome and extends from inter-patient variability and intra-tumoral cellular diversity to the variety of cellular interactions in the tumor microenvironment (TME) (Sottoriva et al., 2013; Patel et al., 2014; Yabo et al., 2022). Until recently, characterization of GBM tumors relies on bulk tumor subtyping and histopathological traits (Verhaak et al., 2010; Wen et al., 2020); however, limitations exist due to the lack of cellular resolution. The advent of single-cell sequencing methodologies now allows clinicians and scientists to discern differences between individual cells from a genomic, epigenomic, transcriptomic, and/or proteomic perspective, allowing for a deeper characterization of the cellular variability within GBM. Furthermore, the emergence of spatially resolved omics technologies, which provide geographical data within tissue samples at cellular resolution, allows for detailed interrogation of GBM tumor cells and their spatial arrangement while preserving tumor niches.

In this review, we cover recent findings regarding cellular diversity in time and space as well as the arsenal of spatially resolved omics approaches available that set the stage for deep exploration of GBM heterogeneity and temporal evolution. Furthermore, we discuss the utilization of these technologies in unveiling novel tumor mechanisms and molecular targets that have the potential to be translated into clinical therapeutics.

### Single-cell characterization of GBM cellular states

Prior to the development of single-cell omics, GBM tumor classification consisted of molecular subtypes based on bulk genomic and transcriptomic tumor profiles (Verhaak et al., 2010). These molecular subtypes, characterized by Verhaak et al., provided a way to subset patient tumors and identify common tumor phenotypes associated with treatment response and survival (Verhaak et al., 2010). However, this approach defined tumors by their average overall profile, which overlooks individual cell phenotypes and dynamic cellular transitions. In the last decade, advancement in sequencing technologies have allowed researchers to analyze tumors at single-cell resolution, unveiling new dimensions to the already vast degree of cellular heterogeneity we see in GBM. Early studies demonstrated a co-existence of the standard molecular subtypes within individual tumors as well as extensive inter-patient variability (Patel et al., 2014; Wang et al., 2017). While all tumors had a dominant molecular subtype - proneural, classical, or mesenchymal - which correlated to the assigned bulk classification, each tumor also had heterogeneous representation of the other subtypes with some individual cells expressing signatures for more than one subtype, termed “hybrid” cells (Patel et al., 2014). Complementary studies showcase the capacity of GBM cells to transition between subtypes (Wang et al., 2017; Varn et al., 2022; Hoogstrate et al., 2023), showing that increased representation of the classical and/or mesenchymal signatures in proneural tumors significantly worsened patient survival (Patel et al., 2014; Wang et al., 2017) These studies highlight the clinical importance of understanding tumor heterogeneity in GBM.

**FIGURE 1:**
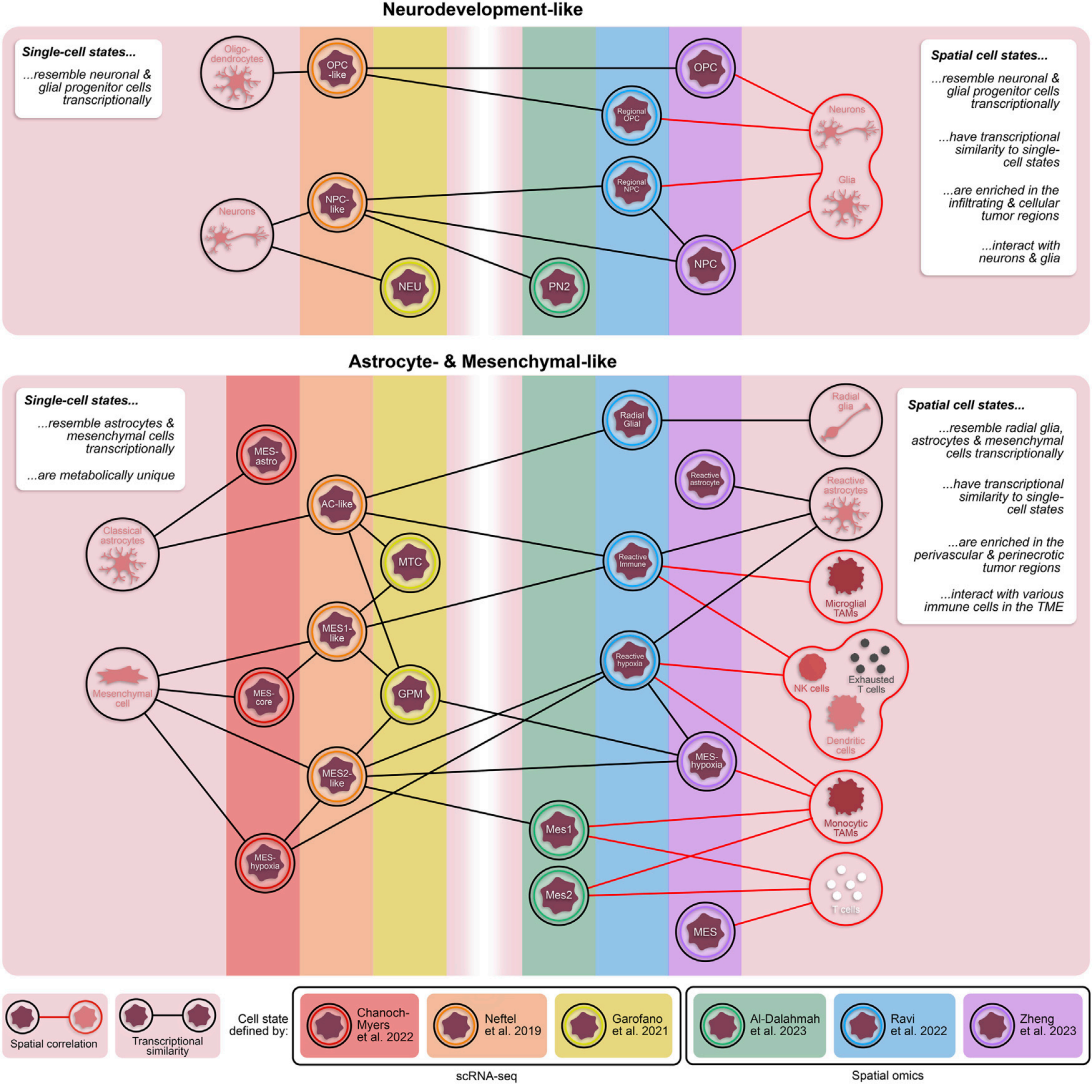
Transcriptionally and spatially distinct cellular states in GBM. Single-cell and spatially resolved technologies have revealed vast heterogeneity in tumor cell states within patient GBMs. While each of these states are fundamentally unique, there are cross-study similarities in their transcriptional profiles. Additionally, cellular states defined through spatial omics approaches have some transcriptomic overlap with states defined by dissociative single-cell techniques. The inclusion of spatial technologies in the study of heterogeneous GBM cell states provides valuable information about proximity of tumor cell states to other cell types, including non-neoplastic cells in the tumor microenvironment. TAMs = tumor-associated monocytes; NK cells = natural killer cells.

In recent years, our molecular understanding of GBM has grown substantially with several studies defining novel, transcriptionally distinct cellular states (Figure 1) (Neftel et al., 2019; Couturier et al., 2020; Garofano et al., 2021; Chanoch-Myers et al., 2022). For example, Neftel et al. described four transcriptionally and genetically unique cellular states (OPC-, NPC-, AC-, and MES-like) that demonstrated significant state plasticity in murine xenograft models (Neftel et al., 2019). Another study by Garofano et al. classified cell states along two axes, neurodevelopmental and metabolic, where the two metabolic states, mitochondrial (MTC) and glycolytic/pluri-metabolic (GPM), correlated to patient survival and therapeutic vulnerability (Garofano et al., 2021). Specifically, the GPM state represents a worse prognosis alongside resistance to inhibitors of oxidative phosphorylation, likely attributed to their metabolic versatility. Furthermore, GPM cells and MES-like cells are transcriptionally similar and both express genes involved in myeloid and lymphoid interactions; however, the exact mechanisms of immune modulation, and whether they are immune-activating or -suppressing, were not identified (Neftel et al., 2019; Garofano et al., 2021). A more recent study has expanded the mesenchymal classification by identifying subclasses of the mesenchymal-like state associated with hypoxia (MES-hypoxia) and astrocytic features (MES-astro) (Chanoch-Myers et al., 2022). This study shows that MES-hypoxia cells associate with tumor-associated M2-like macrophage (TAM) abundance and are likely immune-suppressive while MES-astro cells associate with anti-tumor immune activation. These distinctions highlight the significance of the microenvironment in regulating mesenchymal cell-associated immune modulation. Additionally, numerous studies have focused on the highly aggressive, stem-like population of GBM cells (glioma stem cells; GSCs), underscoring their distinctive plasticity and diverse states (Dirkse et al., 2019; Bhaduri et al., 2020; Guilhamon et al., 2021; Richards et al., 2021). These studies demonstrated that GSC states exist along a continuous phenotypic spectrum and can readily transition between various stem-like and/or differentiated states to drive tumorigenesis and enhance tumor heterogeneity (Dirkse et al., 2019; Richards et al., 2021).

As previously mentioned, dynamic cellular plasticity and the resulting heterogeneity in GBM poses a significant challenge for therapeutic development. This inherent adaptability allows GBM tumor cells to efficiently escape current therapies. The underlying mechanisms of resistance, and how they may be circumvented, are not yet fully understood, underscoring the need for additional studies aimed at identifying drivers and their downstream influences on malignancy states. Fortunately, single-cell sequencing studies have already elucidated several mechanistic mediators of GBM cell states associated with anti-tumor immune-suppression and poor prognosis, including environmental conditions (e.g., hypoxia, radiation therapy) and immune interactions, especially tumor cell-TAM interactions. For example, hypoxia serves as a strong stimulus for cellular adaptation of both stem-like and non-stem-like GBM cells by promoting a mesenchymal shift, increasing expression of stemness markers, and disrupting DNA methylation patterning to facilitate state transitions (Joseph et al., 2015; Dirkse et al., 2019; Johnson et al., 2021). Similarly, cell-to-cell interactions between myeloid cells and tumor cells have been shown to regulate cellular transition to a more malignant state (Ye et al., 2012; Hara et al., 2021). Specifically, tumor cell-TAM interactions increase invasiveness of GSCs and induce a mesenchymal shift in tumor cells through TGF-β signaling and oncostatin receptor-ligand interaction, respectively (Ye et al., 2012; Hara et al., 2021). Furthermore, epigenetic alterations, including dysregulation of DNA methylation and chromatin modifiers, and various transcription factors have been implicated in determining cellular states and transitions (Chaligne et al., 2021; Guilhamon et al., 2021; Johnson et al., 2021; Ma et al., 2021). CRISPR-based screening revealed that SP1 is necessary for maintaining GSCs involved in the immune response while FOXD1 is critical for GSCs associated with angiogenesis (Guilhamon et al., 2021), highlighting potential state-specific therapeutic targets that warrant further investigation.

Beyond identifying drivers of cellular states and transitions, understanding the resultant impact on the brain microenvironment, tumor progression, therapy response and patient prognosis is crucial. Numerous attempts have been made at characterizing the relationships between transcriptionally distinct tumor cell states and immune cells in the TME using single-cell RNA-sequencing (scRNA-seq), revealing strong associations between mesenchymal-like GBM cell states, immune-suppressive macrophages, and dysfunctional anti-tumor T cells (Wang et al., 2017; Yuan et al., 2018; Chanoch-Myers et al., 2022; Xiao et al., 2022). Cell-cell interactions are difficult to discern using dissociative techniques where spatial context is absent, and these studies, therefore, must rely on imperfect analyses such as ligand-receptor pair analyses (Yuan et al., 2018; Xiao et al., 2022) and/or inferred cell abundances based on deconvolution of bulk sequencing data (Wang et al., 2017; Chanoch-Myers et al., 2022). These approaches predict interactions solely based on gene expression profiles, which can be beneficial for hypothesis generation, but are incapable of assessing cell proximity, a critical component for most cell-cell interactions. Cytometry time-of-flight (CyTOF) analysis, which uses an antibody panel to label dissociated cells for subset identification, has been utilized to study the immune tumor microenvironment in GBM (Friebel et al., 2020; Fu et al., 2020; Lee et al., 2021; Simonds et al., 2021). However, these studies focus only on immune cells and, therefore, do not afford the opportunity to explore relationships with the malignant cell population.

Single-cell sequencing has also identified cell states associated with patient prognosis and therapeutic response in GBM. For example, a subset of mesenchymal tumor cells and an invasive subset of GSCs have both been correlated with decreased patient survival (Guilhamon et al., 2021; Chen X. et al., 2022) while tumor cells that depend on mitochondrial function are associated with longer survival (Garofano et al., 2021). Moreover, several studies have identified unique therapeutic vulnerabilities in subsets of malignant cells identified by scRNA-seq (Bhaduri et al., 2020; Couturier et al., 2020; Garofano et al., 2021; Richards et al., 2021). These include a subset of astrocyte-like cells that preferentially utilize mitochondrial metabolism and are therefore susceptible to oxidative phosphorylation inhibitors (Garofano et al., 2021), chemoresistant progenitor GSCs that are vulnerable to inhibition of the transcription factor E2F4 (Couturier et al., 2020), and GSCs associated with injury response mechanisms that are uniquely targetable by knocking out inflammatory response genes (e.g., ITGB1, ILK, and WWTR1) (Richards et al., 2021). As demonstrated by these studies, using single-cell technology to examine how cell states relate to prognosis and therapy response has been more informative to date than analyzing interactions with the immune TME which is more highly dependent on spatial relationships. However, based on the knowledge that environmental interactions regulate cellular states and adaptability, spatial context will provide another dimension critical for comprehensively identifying clinically translatable cellular and molecular targets.

### Spatially resolved technologies for GBM

Unlike single-cell approaches that rely on cell dissociation, spatially resolved technologies maintain tissue architecture and allow for in-depth exploration of tissue heterogeneity and cellular phenotypes while preserving spatial context. Currently, a variety of approaches are available that widely range in their spatial resolution, target depth, and target molecule(s) (Table 1). These can be subdivided based on their fundamental mechanism into spatial barcoding, *in situ* sequencing, and *in situ* imaging. Spatial barcoding, which involves sequencing of oligo-conjugated molecules that bind to mRNA transcripts in the tissue, is arguably the most commonly used subclass of spatial transcriptomics techniques. Examples of spatial barcoding platforms include 10X Visium (standard or CytAssist), Slide-Seq (Xiong et al., 2022), and high-definition spatial transcriptomics (HDST) (Vickovic et al., 2019), Stereo-seq (Chen A. et al., 2022), and Seq-Scope (Cho et al., 2021). These techniques are especially useful in discovery-based, hypothesis-generating studies since they cover virtually the entire transcriptome. While spatial barcoding is advantageous for this unbiased transcriptome coverage, the data is collected at multi-cellular spot-wise resolution (up to 100 microns), where one data point averages numerous cells, which requires computational deconvolution to determine the cellular composition of each spot. Sample-matched scRNA-seq is therefore complementary to spatial transcriptomics because it facilitates the spatial deconvolution of data (Ma and Zhou, 2022). Notably, several spatial barcoding techniques, including HDST, Stereo-seq, and Seq-SCOPE, are high resolution and capture spots at subcellular size (0.5–2 microns), thereby foregoing the need for spatial deconvolution. These approaches have yet to be used in GBM studies, likely due to technical challenges and strict instrument requirements (Wang Y. et al., 2023).

**TABLE 1:** Spatially resolved technologies for studying GBM.

Technique	Class	Resolution	No. of detectable Targets	Target Molecule(s)	Refs in GBM
10X Visium	Spatial Tx	50 um	Whole transcriptome^a^	mRNA	Ravi et al. (2022a), Ravi et al. (2022b), Xiong et al. (2022), Yabo et al. (2022), Al-Dalahmah et al. (2023), Jain et al. (2023), Liu et al. (2023), Ren et al. (2023), Sattiraju et al. (2023), Zheng et al. (2023)
10X Visium CytAssist	Spatial Tx	50 um	Whole transcriptome^a^, ≤31 proteins	mRNA + Protein	
Slide-seq (Xiong et al., 2022)	Spatial Tx	10 um	Whole transcriptome	mRNA	
HDST (Vickovic et al., 2019)	Spatial Tx	2 um	Whole transcriptome	mRNA	
Stereo-seq (Chen et al., 2022b)	Spatial Tx	0.5-1 um	Whole transcriptome	mRNA	
Seq-scope (Cho et al., 2021)	Spatial Tx	0.6 um	Whole transcriptome	mRNA	
Nanostring GeoMx (Merritt et al., 2020)	Spatial Tx	≤600 um^b^	Whole transcriptome, <580 proteins	mRNA + Protein	Wang et al. (2022), Kim et al. (2023), Loussouarn et al. (2023), Moffet et al. (2023)
Spatial CITE-seq (Liu et al., 2023)	Spatial Tx	25 um	Whole transcriptome, ≤270 proteins	mRNA + Protein	
FISSEQ (Lee et al., 2015)	*In situ* sequencing	Subcellular	Whole transcriptome	mRNA	
STARmap (Wang et al., 2018)	*In situ* sequencing	Subcellular	Whole transcriptome	mRNA	
seqFISH (Eng et al., 2019)	*In situ* imaging	Subcellular	≤10 K genes	mRNA	
Vizgen MERSCOPE	*In situ* imaging	Subcellular	≤500 genes	mRNA	
10X Xenium	*In situ* imaging	Subcellular	≤500 genes	mRNA	Moffet et al. (2023)
Nanostring CosMx (He et al., 2022)	*In situ* imaging	Subcellular	≤6000 genes, ≤68 proteins	mRNA + Protein	Moffet et al. (2023)
IMC (Giesen et al., 2014)	*In situ* imaging	Subcellular	≤40 proteins	Protein	Ravi et al. (2022a), Ravi et al. (2022b), Karimi et al. (2023), van Hooren et al. (2023)
CODEX (Goltsev et al., 2018)	*In situ* imaging	Subcellular	≤100 proteins	Protein	Shekarian et al. (2022)
MSI (e.g., MALDI-MSI) (Taylor et al., 2021)	*In situ* imaging	Subcellular	Variable	Proteins, Lipids, Metabolites, Drugsetc.	Ravi et al. (2022a), Coy et al. (2022), Duhamel et al. (2022), Rashidi et al. (2024)

^a^ Up to 18,000 unique genes

^b^ Can go as small as 10 um but Nanostring recommends at least 20 cells per region of interest due to analytical challenges; Tx, transcriptomics.

An extension of the 10X Visium platform, Visium CytAssist, provides the same transcriptomic data with the added advantage of protein detection using oligo-tagged antibodies. Another spot-wise dual spatial transcriptomic and proteomic technique growing in use is the Nanostring GeoMx platform (Merritt et al., 2020). This platform uses photocleavable oligo-labeled probes and/or antibodies that target mRNA and protein, respectively, that are selectively released from the tissue regions of interest (ROIs) and sequenced. While the resolution is lower, Nanostring allows for supervised selection of ROIs based on preliminary tissue staining, allowing for the analysis of both the transcriptomic and proteomic profiles of specific tissue regions. Spatial CITE-seq, a recently developed technique that evolved from the previous single-cell CITE-seq approach, allows for simultaneous detection of both whole transcriptome and up to 270 proteins with a 25-micron resolution (Liu et al., 2023). Similar to Nanostring GeoMx, spatial CITE-seq involves oligo-labeled mRNA probes and protein-specific antibodies. These oligo barcodes are subsequently sequenced for transcript and protein identification. Having just emerged, spatial CITE-seq has not yet been used in the setting of GBM but is a promising new technology for multi-omic spatial exploration. In comparison to transcriptomic approaches, these dual transcriptomic and proteomic technologies provide an additional dimension of data that can be used to analyze relationships between transcriptional states and protein-defined cell types. However, validation studies are necessary for robust conclusions due to the multi-cellular resolution of these approaches and consequential computational complexities.

To achieve higher spatial resolution, *in situ* approaches can be employed. Generally, these technologies afford subcellular investigation of mRNA and/or protein expression with the drawback of limited target depth compared to spatial barcoding technologies. *In situ* sequencing, such as FISSEQ (Lee et al., 2015) and STARmap (Wang et al., 2018), provides a greater target depth than *in situ* imaging, covering most of the transcriptome, but run into issues with instrument limitations and optical crowding (i.e., indistinguishable fluorescent signals due to overlap) (Kleino et al., 2022). These methods can extensively define the spatially relevant transcriptional profile of individual cells within tissue, outperforming spatial barcoding in regard to resolution and fluorescent-based *in situ* imaging in terms of target depth. Transcriptomic imaging techniques involving fluorescence *in situ* hybridization (FISH) include seqFISH (Eng et al., 2019), Vizgen MERSCOPE, and Nanostring CosMx (He et al., 2022). The newest of these, MERSCOPE and Nanostring CosMx, are both commercially available. While MERSCOPE can identify up to 500 genes using a customizable probe panel, Nanostring CosMx can analyze up to 6000 genes and 68 proteins simultaneously using either standard or customized fluorescently labeled probes and antibodies, but with a longer imaging time compared to MERSCOPE. The new 10X Genomics platform, Xenium, incorporates both *in situ* sequencing and hybridization by using padlock probes with rolling circle amplification that are subsequently fluorescently labeled and imaged. The successive rounds of fluorescent imaging, fundamental to the Xenium platform, result in a high fluorescent intensity and signal to noise ratio, making it an enticing new option for *in situ* imaging. While *in situ* imaging is limited in the volume of detectable targets, the commercial availability of many of these approaches, especially multi-omic ones, increases their accessibility and reliability. This is consistent with the prevalence of these technologies in recent studies. Furthermore, the subcellular resolution and multi-omic data provided by the CosMx and Xenium platforms yield more conclusive information regarding cellular states, localization, and interactions, relative to spatial barcoding techniques.

Beyond *in situ* transcriptomics, several technologies allow for *in situ* analysis of other molecular targets including proteins, lipids, metabolites, and drugs. For example, imaging mass cytometry (IMC) utilizes metal-tagged antibody probes that are laser ablated and identified using a mass cytometer (Giesen et al., 2014). This provides information on up to 40 proteins at subcellular resolution. Alternatively, CO-Detecting via indEXing (CODEX, now commercially known as PhenoCycler) can detect up to 100 proteins at subcellular resolution through successive rounds of fluorescent-labeled oligo probe hybridization and imaging, similar in context to cyclic immunofluorescence (CycIF) (Lin et al., 2015; Goltsev et al., 2018). Both technologies have flexible protein panel options, permitting customization. A major disadvantage to these approaches is the limit in target depth and the inherent bias in using pre-selected probe panels.

Unbiased spatially resolved proteomics technologies are also available in the form of mass spectrometry imaging (MSI). These methodologies are classified by their ionization method, or method for acquiring tissue analytes, and their mass analyzer which outputs the mass spectrum for each analyte. One commonly used ionization method in cancer studies is matrix-assisted laser desorption ionization (MALDI) (Taylor et al., 2021). Broadly, MSI works by ionizing or removing analytes from the tissue surface and subsequently using a mass spectrometer to identify each analyte. These analytes may include proteins, lipids, metabolites, or drug molecules, depending on the tissue preparation method. In general, MSI-based spatial approaches provide subcellular resolution of detectable tissue analytes with the disadvantage of complex downstream analysis and the need for complementary technologies in order to identify target analytes, depending on their molecular class. MALDI is advantageous over other ionization methods given the larger variety of detectable molecular classes, pixel resolution, and types of useable tissue (e.g., fresh-frozen, FFPE, etc.), and is particularly useful for proteomics, lipidomics, and/or metabolomics studies.

Collectively, the large variety of spatially resolved technologies currently available has the capacity to provide an abundance of insight into GBM biology, with each approach having unique utility in spatial studies. Importantly, the selection of analytic tools for spatial omics is also expanding and includes options for neighborhood analyses, which analyze gene expression patterns, cell proximities, and ligand-receptor interactions to explore cell interactions (Yuan and Bar-Joseph, 2020; Dries et al., 2021; Pham et al., 2023), and spatiotemporal trajectory analyses, which can infer cell state transitions and progressions relative to space (Pham et al., 2023). In the broad field of cancer biology, the aforementioned technologies have granted revolutionary insight into the dynamic and heterogeneous nature of cancers. In particular, spatial omics have unveiled spatial diversity of cancer cell states, invasive progression of tumors, spatially distinct cellular transitions in response to stimuli, cellular interactions within the TME, and tissue localization of treatment-resistant cancer cells (Zhou et al., 2023).

### Spatiotemporal tumor cell dynamics in GBM

#### Spatially distinct cellular and tissue states

**FIGURE 2:**
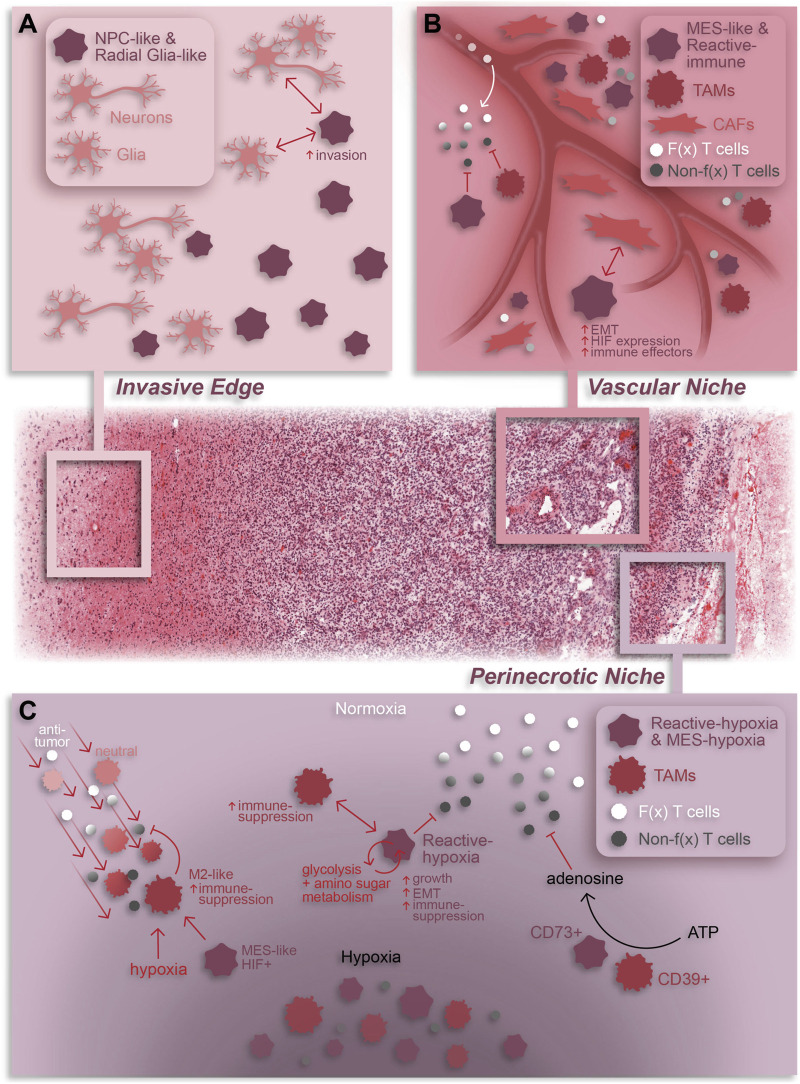
Summary of cellular dynamics and localization in GBM based on spatial omics. The GBM tumor microenvironment is characterized by regional niches (invasive edge, cellular tumor, vascular niche, and perinecrotic niche) enriched with a variety of cell types and interactions that perpetuate or result from the niche-specific environmental conditions. **(A)** The invasive edge of GBM is characterized by normal brain cells (e.g., neurons and glia) and infiltrating tumor cells. In general, these tumor cells are NPC-like and/or Radial glia-like and interact with normal brain cells to increase invasion and promote tumor progression. **(B)** The vascular niche is phenotypically diverse tumor region composed of infiltrating anti-tumor lymphocytes, an abundance of pro-tumor TAMs and CAFs, and mesenchymal-like and immune-modulating (reactive immune) tumor cells. Within this niche, CAF-GSC interactions promote an aggressive tumor cell phenotype while immune-suppressive TAMs and tumor cells work together to repress anti-tumor T cell function **(C)** The perinecrotic or hypoxic niche is a highly immune-suppressive tumor region characterized by an abundance of hypoxia-responsive, immunosuppressive tumor cells and tumor cell-TAM interactions. TAMs are recruited to the perinecrotic niche by tumor cells and, broadly, hypoxic conditions where they undergo a GBM cell-mediated pro-tumor transformation to an M2-like, immune-suppressive state. Reciprocally, TAMs support the growth of hypoxic tumor cells and drive them to a mesenchymal-like, immune-suppressive state. Additionally, TAMs and tumor cells cooperate to repress anti-tumor T cell function through adenosine signaling. Notably, anti-tumor T cells are also suppressed by hypoxia in general. F(x) = functional; Non-f(x) = non-functional; EMT = epithelial-to-mesenchymal transition. The H&E-stained tissue in this graphic was obtained from the Ivy GAP database (tumor tissue sub-block W2-1-1-F.1.01).

Prior to high resolution spatial technologies, sequencing insight into tissue architecture was only achievable by conducting bulk sequencing on tumor specimens that had been micro sampled based on pre-defined histopathological regions. The Ivy Glioblastoma Atlas Project collected over 100 samples from histopathological tumor regions for RNA-sequencing, providing unparalleled insight, at the time, into niche-specific transcriptional and genetic profiles in GBM (Puchalski et al., 2018). In particular, this project identified signaling pathways unique to each niche, such as cellular stress response and inflammatory response in the perinecrotic region and cell migration and immune activation in the perivascular region. While this data continues to be useful today, the emergence of high resolution spatially resolved sequencing tools allow for detailed and in-depth analysis of tumor tissue at cellular resolution. Going beyond the classically defined histopathological regions in GBM, spatial technologies have uncovered a vast degree of knowledge relating to the localization and co-localization of malignant and non-malignant cells, and their related interactions within specific niches (Table 2; Figure 2).

**TABLE 2:** Publicly available primary spatial datasets from GBM patients.

Author and year	Target Molecule(s)	Spatial Tool(s)	Single-cell Tool(s)	Data storage
Moffet et al. (2023)	mRNA	Nanostring GeoMx and CosMx, Xenium	-	GSE232469 (GeoMx), Mendeley Data doi: 10.17632/wc8tmdmsxm.2 (CosMx, Xenium)
Al-Dalahmah et al. (2023)	mRNA	Visium	snRNA-seq	GSE228500
Ren et al. (2023)	mRNA	Visium	-	GSE194329
Wang et al. (2022)	mRNA, Proteins	Nanostring GeoMx	snRNA-seq	GSE174554
Duhamel et al. (2022)	Proteins	MALDI-MSI	-	ProteomeXchange Consortium PXD016165
Coy et al. (2022)	Metabolites, Proteins	MALDI-MSI, CycIF	-	NMDR ID: PR001406 (MALDI), synapse.org syn30803310 (CyCIF)
Shekarian et al. (2022)	Proteins	CODEX	-	Provided in supplemental data
Ravi et al. (2022a)	mRNA, Proteins, Metabolites	Visium, IMC, MALDI-MSI	scRNA-seq	Data Dryad: https://doi.org/10.5061/dryad.h70rxwdmj
Chen et al. (2022a)	mRNA	-	scRNA-seq	HRA002610
Xiao et al. (2022)	mRNA	-	scRNA-seq	GSE135045
Chaligne et al. (2021)	mRNA, DNA methylation	-	scRNA-seq, scRRBS	GSE151506
Johnson et al. (2021)	mRNA, DNA methylation	-	scRNA-seq, scRRBS	Synapse.org/singlecellglioma
Garofano et al. (2021)	mRNA	-	scRNA-seq	Synapse.org synID: syn22314624
Richards et al. (2021)	mRNA	-	scRNA-seq	EGAS0001004645 or via singlecell.broadinstitute.org
Couturier et al. (2020)	mRNA	-	scRNA-seq	EGAS00001004422
Bhaduri et al. (2020)	mRNA	-	snRNA-seq	SRP132816 or via cells.ucsc.edu/?ds = gbm
Neftel et al. (2019)	mRNA	-	scRNA-seq	GSE131928 or via singlecell.broadinstitute.org

snRNA-seq, single-nucleus RNA-seq; scRRBS, single-cell reduced-representation bisulfite sequencing.

In a pioneering study, Ravi et al. combine spatial transcriptomics, MALDI-MSI, and IMC to describe five spatially and transcriptionally distinct malignant states in patient tumors–regional oligodendrocyte progenitor cell-like (OPC), regional neuronal progenitor cell-like (NPC), radial glia, reactive immune, and reactive hypoxia (Ravi et al., 2022a). Aside from their transcriptional profiles, these states differ in their metabolic profiles, chromosomal variations, and co-localization with non-neoplastic cell types. Notably, both reactive immune and reactive hypoxia are associated with increased TAM and T cell abundance, especially PD1+ T cells, demonstrating that these states reside in regions of immune infiltration (Figure 2C). Additionally, the reactive hypoxia state utilizes glycolysis and amino sugar metabolism more so than other states. Given the dependence of anti-tumor lymphocytes on glucose metabolism (Ho et al., 2015; Siska et al., 2016), this hints at a potential immune-suppressive role of reactive hypoxia cells which may monopolize glucose in the TME.

A subsequent study conducted by Zheng et al. identified five spatially distinct tumor cell states–NPC, OPC, reactive astrocyte, mesenchymal-like (MES), and a subset of MES cells termed MES-hypoxia (Zheng et al., 2023). In line with the previous study, tumor cell states tend to fall under one of two umbrellas: neurodevelopment or astrocyte/mesenchymal-like. In comparison, both the NPC and OPC states from Zheng et al. resembled the regional NPC and spatial OPC states from Ravi et al., respectively, while both the reactive astrocyte and MES states had overlap with the reactive immune state (Figure 1). Notably, the MES-hypoxia state, which closely resembles the reactive hypoxia state, is associated with a worse prognosis in patients. While there are transcriptional similarities between these spatial cell states and the cell states previously defined by scRNA-seq (Figure 1) (Ravi et al., 2022a; Al-Dalahmah et al., 2023; Zheng et al., 2023), these spatially distinct states are fundamentally characterized with respect to their local microenvironment and are therefore influenced by surrounding cells and nutrients, providing an added dimension critical for exploring tumor-immune interactions.

Spatially resolved omics have also helped elucidate unique tissue regions, composed of both neoplastic and non-neoplastic cell types, within GBM patient specimens. Ren and colleagues described four tissue regions–tumor core, invasive niche, vascular niche, and hypoxic niche - using spatial transcriptomics that corresponded to enrichment of specific cell states (Ren et al., 2023). For example, astrocyte-like and radial glia-like GBM cells localized in the invasive niche while OPC-like GBM cells were more prominent in the tumor core suggesting that astrocyte-like and radial glia-like cells may be more involved in tumor invasion and progression (Figure 2A). A study by Al-Dalahmah et al. also characterized distinct tissue states using spatial transcriptomics. Of the three identified states, two were somewhat homogeneous in composition with one predominantly enriched in non-neoplastic brain cells and the other predominantly enriched in CNV-positive tumor cells, reminiscent of the cellular tumor niche (Wen et al., 2020). A more heterogeneous third state was enriched for astrocyte/mesenchymal-like tumor cells, reactive astrocytes, macrophages, and T cells and was associated with shortened patient survival (Al-Dalahmah et al., 2023), likely representing aspects of both the perivascular and perinecrotic niches (Wen et al., 2020). In another study, a combination of Nanostring GeoMx, CosMx and Xenium elucidated heterogeneous cell neighborhoods in GBM. Two major niches conserved across patients and spatial platforms were a brain-intrinsic environment, marked by astrocytic- and oligodendrocytic-like tumor cells and microglia, and a brain-extrinsic niche consisting of peri-vascular enrichment of mesenchymal-like cells, monocytes, T cells, and neutrophils (Figure 1) (Moffet et al., 2023).

Aside from transcriptomics, distinct tissue regions have also been uncovered using spatial proteomics. By using a combination of MALDI-MSI proteomics and shotgun proteomics, Duhamel et al. described three unique tissue regions (Duhamel et al., 2022). These regions were predominantly distinct from histopathological niches, though with some similarities. One region resembled both the perinecrotic and perivascular niches and had increased expression of immune-related proteins while another region embodied traits of both the perivascular niche and cellular tumor and was enriched for tumorigenic proteins. The third included both infiltrating tumor and cellular tumor and expressed neurodevelopmental and synaptic transmission proteins. Interestingly, these proteomic tissue states appear to align, molecularly, with the transcriptional tissue states defined by Al-Dalahmah et al.; however, a direct comparison has not been done. Notably, several region-specific proteins were determined to be prognostic markers for patients. In particular, ANXA11, a protein involved in tumor proliferation and invasion in other cancers (Liu et al., 2015; Hua et al., 2018), was highly expressed in the neurodevelopmental region and correlated with poor patient prognosis. A separate study by Shekarian et al. unveiled seven distinct tissue states via CODEX spatially resolved proteomics technology (Goltsev et al., 2018; Shekarian et al., 2022). Researchers demonstrated enrichment of an adaptive immune state, characterized by infiltrating lymphocytes and M2-like macrophages, and two vascular-related states in the tumor core and tumor periphery, respectively. Additionally, they determined that the tumor core has increased cellular density and heterogeneity compared to the more homogeneous cellular composition in the tumor periphery. Given what is known about the effect of TAM interactions on tumor cell plasticity (Ye et al., 2012; Hara et al., 2021), one can predict that this tumor core heterogeneity may result from increased interactions facilitated by higher immune cell infiltration and cellular density.

#### Insights into cell interactions

Spatially resolved omics can also provide insight into the diversity and consequences of cell-cell interactions within tumor regions. Spatial transcriptomics and proteomics have associated invading brain tumor cells with non-neoplastic glial cells in the invasive tumor edge (Wang et al., 2022). Spatial transcriptomics have also shown that cancer-associated fibroblasts (CAFs), a subset of non-neoplastic tumor-resident fibroblasts, also co-localize with tumor cells and support GBM progression (Jain et al., 2023). Specifically, CAFs are in close proximity to mesenchymal-like and stem-like GBM cells, endothelial cells, and TAMs within the perivascular niche (PVN) (Figure 2B). Furthermore, CAFs are recruited to the PVN by GSCs where they promote a malignant GSC phenotype characterized by HIF1a activation, epithelial-to-mesenchymal transition and increased cell proliferation. Notably, intracranial implantation of CAFs with GSCs in mice enhanced tumorigenesis relative to tumors formed by GSCs alone, emphasizing the potent pro-tumoral effects of CAFs in GBM.

Regarding the immune TME, spatial transcriptomics and proteomics have shown that tumor cells co-localize with both exhausted CD8^+^ T cells (Ravi et al., 2022b) and a variety of myeloid cells (Coy et al., 2022; Shekarian et al., 2022; Al-Dalahmah et al., 2023; Jain et al., 2023; Sattiraju et al., 2023). Interactions between tumor cells and myeloid cells have a well-established role in promoting an immune-suppressive TME and multiple scRNA-seq studies have revealed mechanisms whereby TAMs can drive a mesenchymal transition in GBM tumor cells (Hara et al., 2021; Xiong et al., 2022). One study using spatial transcriptomics and metabolomics revealed that activated TAMs surrounding the hypoxic niche produce creatine to support the growth of nearby tumor cells in an otherwise low-nutrient environment, highlighting the importance of TAM interactions for tumor cell survival (Figure 2C) (Rashidi et al., 2024). Conversely, a spatial transcriptomics study demonstrated that hypoxic tumor cells promote an immune-suppressive phenotype in TAMs through induction of CCL8 and IL1B cytokines (Figure 2C) (Sattiraju et al., 2023). An additional spatial study based on MALDI-MSI and CycIF discovered that co-localization of CD39^+^ myeloid cells and CD73^+^ tumor cells results in increased extracellular adenosine, an immune-regulatory molecule (Figure 2C) (Coy et al., 2022). Notably, CD73 expression in tumor cells is correlated to HIF1a expression and enriched in the perinecrotic niche. This link between the tumor cell hypoxic response and immune-suppression is corroborated by evidence that HIF1a activation in tumor cells results in a mesenchymal shift and expression of immunosuppressive genes and is linked with poor patient survival and tumor recurrence (Joseph et al., 2015; Sattiraju et al., 2023). In this same study, Sattiraju and colleagues elegantly showed that adaptive immunity is necessary for generation of hypoxic tumor zones which in turn work to suppress the anti-tumor immune response. Furthermore, obstructing communication between hypoxic tumor cells and TAMs reduces hypoxia frequency, suggesting that generation of hypoxic regions is at least partially dependent upon tumor-TAM interactions. Together, these data highlight the pro-tumor impact of immune infiltration and hypoxic conditions on anti-tumor immunity and tumor cell phenotypes.

#### Spatiotemporal TME changes in response to therapy

Based on the inevitability of tumor recurrence and high degree of inherent plasticity in GBM, understanding how the spatial landscape of GBM changes over time and in response to therapy will go a long way toward advancing therapeutic development for recurrent GBM. A study by van Hooren et al. revealed an increase in monocyte-derived macrophages (MDMs) at time of recurrence in GBM (van Hooren et al., 2023). Van Hooren et al. used spatially resolved IMC to demonstrate MDM, regulatory T cell (Treg) and PD1-high CD8^+^ T cell enrichment in recurrent GBM, resulting in increased MDM-CD8^+^ T cell and Treg-CD8^+^ T cell interactions and enhanced immune-suppressive activity. Spatial changes in response to immunotherapy, specifically anti-CD47 and anti-PD1, have also been explored by Shekarian et al. In their study, treatment of patient tumor explants with single or combinatorial immunotherapy resulted in increased CD4^+^ and CD8^+^ T cell infiltration and M1-like macrophage presence, as determined by CODEX spatial technology (Shekarian et al., 2022). Immunotherapy-treated explants had markedly high levels of interferon-gamma, especially those treated with anti-PD1, suggesting functional activation of infiltrating lymphocytes. Notably, explants with low interferon-gamma levels post-treatment had a higher abundance of M2-like macrophages and enhanced expression of checkpoints on T cells, suggesting that a prevalence of immune-suppressive TAMs may impair immunotherapy-induced T cell activation. While valuable, particularly in studying immune responses to immunotherapy treatment, these studies omitted analysis of the effects on the neoplastic cell populations. Given what we know about tumor cell and TME interactions impacting tumorigenesis and immune regulation, improving our understanding of tumor cell phenotype transitions in the context of neighboring non-neoplastic cells will be crucial for development of effective therapeutics.

### Therapeutic perspectives

GBM tumors are notoriously resistant to current immunotherapies with clinical attempts continuing to fall short of therapeutic efficacy endpoints (Medikonda et al., 2021). A multitude of therapeutic modalities that have seen success in other cancers including immune checkpoint inhibitors (ICIs), CAR-T cells, vaccines, and oncolytic viruses have been tested in GBM clinical trials but fail to significantly affect patient survival. Despite several mechanisms of immunotherapy resistance being proposed (Wang et al., 2021), our understanding of tumor-immune cell interaction in GBM remains inadequate. Combining single-cell transcriptomic and spatial technologies provides an opportunity to robustly study multiple facets of the interactions between GBM cells and the microenvironment, particularly when used in tandem (Ravi et al., 2022a; Wang et al., 2022). Spatially resolved multi-omics present immeasurable potential in GBM that has only just breached the surface. Expanding our understanding of spatiotemporal cell dynamics in GBM will advance therapeutic development. In the final section of this review, we provide a clinical perspective on the findings discussed previously, highlighting current pre-clinical and clinical therapeutic advances leveraging the technologies discussed herein.

#### Inhibiting pro-tumor cell communication

**FIGURE 3:**
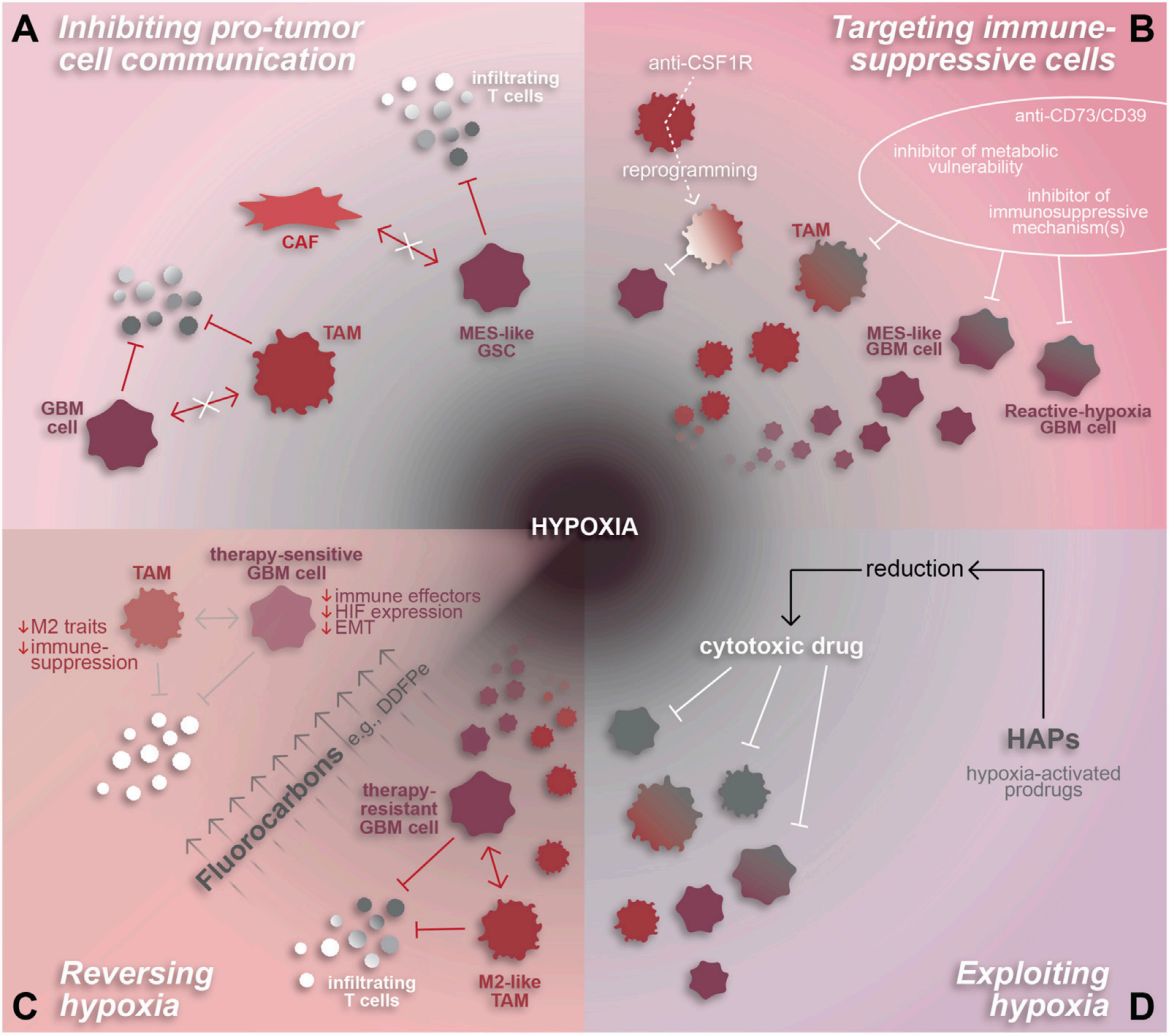
Hypoxia-related therapeutic targets in GBM. The pro-tumor and highly immune-suppressive effects of hypoxia make it an attractive therapeutic target for GBM. **(A)** Hypoxia-associated cell interactions that inhibit the anti-tumor immune response by inducing immune-suppressive phenotypes in GBM cells and/or TAMs can be attenuated by inhibiting their mechanisms of intercellular communication. **(B)** Immune-suppressive cell states can also be targeted directly through various approaches including anti-CD73/CD39 or inhibitors designed to target metabolic vulnerabilities and/or specific immune-suppressive mechanisms or effectors. TAM-specific agents, such as anti-CSF1R, can be used to reprogram TAMs to an anti-tumor state. **(C)** Reversal of hypoxia is another promising therapeutic approach, whereby fluorocarbons have been shown to reduce hypoxia and resensitize GBM cells to chemoradiation. Hypothetically, hypoxia reversal has the potential to reduce the pro-immunosuppressive effects of hypoxia on TAMs and GBM cells and reduce T cell repression to enhance the anti-tumor immune response. **(D)** Alternatively, hypoxia-activated prodrugs are a method of exploiting hypoxic conditions in order to chemically reduce and thereby activate cytotoxic drugs to target malignant and pro-tumor cells within the hypoxic niche.

One potential avenue for augmenting efficacy of immunotherapy in GBM involves restricting tumor-supportive cell-cell interactions, especially those that induce immunosuppressive phenotypes. For example, spatial analyses have demonstrated both CAF-tumor cell and TAM-tumor cell interactions that promote tumor progression and immune-suppression in association with hypoxia (Coy et al., 2022; Jain et al., 2023; Sattiraju et al., 2023). As mentioned in previous sections, these interactions involve various signaling mechanisms and involve GBM cells residing in the hypoxic niche. CAF-mediated induction of a hypoxic response, mesenchymal shift, and expression of immune-suppressive genes in GSCs relies on osteopontin and HGF signaling (Jain et al., 2023). Alternatively, tumor cell-TAM interactions can involve TGF-b and oncostatin signaling or creatine metabolism to support immunosuppressive TAMs and tumor cells, respectively (Ye et al., 2012; Hara et al., 2021; Rashidi et al., 2024). Inhibiting these signaling mechanisms would be expected to decrease immune-suppression and potentially increase anti-tumor T cell infiltration and/or functionality (Figure 3A).

#### Targeting immune-suppressive cells

Another approach to boost the anti-tumor immune response is to target immunosuppressive cell types directly (Figure 3B). Malignant cell states that modulate the immune TME, such as reactive hypoxia (Ravi et al., 2022a) or MES-hypoxia (Zheng et al., 2023) cells which express immune-suppressive factors and correlate spatially with immune-suppressive TAMs, are a promising therapeutic target. Approaches to effectively inhibit these states remain unclear. One option could be to identify and target molecular pathways downstream of the hypoxic response, such as dysregulated DNA methylation, the mesenchymal shift, cytokine signaling in TAMs, or molecular drivers of the reactive hypoxia or MES-hypoxia tumor cell states (Joseph et al., 2015; Johnson et al., 2021; Ravi et al., 2022a; Chanoch-Myers et al., 2022; Sattiraju et al., 2023). Similarly, data on the unique metabolic profiles identified using MALDI-MSI (Ravi et al., 2022a) may be leveraged to identify targetable metabolic vulnerabilities in hypoxic GBM. Another option would be to target potent immunosuppressive effectors individually, such as with a CD73 inhibitor like α,β-methylene-ADP (APCP) (Cho et al., 2006), or collectively by inhibiting a common upstream driver like TGF-β signaling (Johnson ALJK et al., 2022). As previously mentioned, spatial proteomics revealed that tumor cell expression of CD73 is strongly correlated to TAM co-localization, hypoxia, and immune-suppression. Therefore, inhibiting CD73 could impair pro-tumor adenosine signaling in the perinecrotic niche and enhance the anti-tumor immune response.

Due to the abundance of pro-tumor myeloid cells within GBM, especially in recurrent GBM, TAMs have become a novel focus for developing therapies. These attempts have involved direct inhibition of pro-immunosuppressive mechanisms, such as CSF1R, KDM6B, or TREM2 inhibition (Przystal et al., 2021; Goswami et al., 2023; Sun et al., 2023), and TAM reprogramming to an anti-tumor state (Zhang et al., 2016; Chryplewicz et al., 2022; Zhang et al., 2023). Several of these approaches have proven successful in preclinical studies whereby TAM reprogramming increases tumor cell phagocytosis and augments checkpoint inhibitor therapy (Przystal et al., 2021; Chryplewicz et al., 2022; Goswami et al., 2023; Sun et al., 2023). An interesting facet of TAM reprogramming yet to be explored is the impact on tumor hypoxia. Since pro-tumor TAMs are interdependent, both spatially and mechanistically, with hypoxia, one would hypothesize that polarizing TAMs to an anti-tumor state may reduce the immunosuppressive TAM-tumor cell interactions that occur under hypoxic conditions.

#### Reversing or exploiting hypoxia

Another feature of GBM that can be exploited based on spatial understanding is the hypoxic niche. As discussed previously, hypoxia is a potent regulator of cell states in GBM, promoting mesenchymal and stem-like phenotypes in tumor cells and a pro-tumor, immune-suppressive phenotype in TAMs (Joseph et al., 2015; Dirkse et al., 2019; Johnson et al., 2021; Sattiraju et al., 2023). Furthermore, tumor cell states and tumor-immune interactions in the hypoxic niche facilitate tumor growth and suppress anti-tumor immunity (Sattiraju et al., 2023; Rashidi et al., 2024). These tumor-supporting, hypoxia-mediated cell states and mechanisms, discovered using single-cell and spatial technologies, represent new and promising therapeutic opportunities in GBM.

Aside from directly targeting interactions and cell states in the tumor, hypoxic conditions can be therapeutically exploited or reversed using hypoxia-activated cytotoxic agents such as evofosfamide (Evo or TH-302) or agents that increase intra-tumoral oxygen delivery like fluorocarbons, respectively (Teicher et al., 1997; Johnson et al., 2009; Murayama et al., 2012; Anduran et al., 2022) (Figures 3C, D). This would allow for specific targeting of cells residing in hypoxic tumor regions, including immunosuppressive GBM and myeloid cells, in a manner that spares normal brain tissue. Evo has been tested in other cancers where it augmented anti-PD1 therapy in preclinical studies (Jayaprakash et al., 2018; Wang et al., 2023). Translation into clinical trials demonstrated that Evo was well-tolerated and resulted in stable disease status in most patients (Hegde et al., 2021). In GBM, *in vivo* use of Evo −/+ radiation reduced tumor burden, eliminated hypoxia-responsive tumor cells, and reduced the presence of pseudopalisades, a morphological feature indicative of hypoxia surrounding necrotic regions (Sattiraju et al., 2023). Alone, Evo increased vascular density while simultaneously disrupting TAM infiltration and reducing TAM abundance near hypoxia. Beyond the lab setting, a phase II clinical trial tested Evo in combination with bevacizumab, an FDA-approved anti-angiogenic therapy, failed to have a significant impact on overall patient survival compared to a historical control (Brenner et al., 2021). Based on the promising pre-clinical data (Sattiraju et al., 2023), Evo warrants further investigation in the context of GBM, especially in the absence of bevacizumab. Additional studies exploring the specifics of how Evo impacts the anti-tumor immune response and whether it augments GBM immunotherapy could provide valuable insight into methods for targeting hypoxia-mediated immune suppression.

Alternative to exploiting hypoxia, reversing hypoxia via oxygen transporter drugs has been tested in GBM and effectively sensitizes tumor cells to chemoradiation (Teicher et al., 1997; Murayama et al., 2012). This can be achieved through fluorocarbons which efficiently deliver oxygen to hypoxic regions in high volumes through passive diffusion (Johnson et al., 2009). Notably, the fluorocarbon known as dodecafluoropentane emulsion (DDFPe) has been tested against newly-diagnosed and recurrent GBM in clinical trials where it was well-tolerated and effectively reversed tumor hypoxia (Lickliter et al., 2023). Furthermore, the study demonstrated a trend towards increased patient survival and is being tested further in a phase II/III trial [NCT02189109]. The effects of DDFPe have not been explored in pre-clinical GBM studies, highlighting a gap in knowledge with the potential to provide insight into DDFPe’s impact on hypoxia-mediated malignant cell states and suppressed anti-tumor immune response.

The prevalence of immunosuppressive cell types and interactions within and surrounding the hypoxic niche suggests that pinpoint targeting of these cells, pro-tumor interactions, or hypoxia itself may augment the anti-tumor immune response and enhance efficacy of immunotherapy against GBM. A byproduct of disrupted vasculature and consequently decreased oxygen availability, hypoxia is fundamentally dependent on tissue architecture. Therefore, high-resolution spatially resolved technologies, like those discussed in previous sections, are critical to understanding the clinical impact of hypoxia-mediated cell phenotypes and identifying viable therapeutic targets. Multi-omic spatial approaches are especially advantageous in that they allow for in-depth analysis of cell interactions, which can elucidate new mechanisms of hypoxia-mediated anti-tumor immune suppression, further expanding upon those previously identified.

Overall, spatially resolved studies have already revealed several attractive therapeutic targets in GBM (Figure 3). Moving forward, additional spatial approaches paired with robust preclinical testing of novel therapeutics will be critical to advance the field of GBM therapy. Treatments aimed at exploiting hypoxia or targeting hypoxia-mediated immunosuppressive cell states, hold particular promise for GBM and could ultimately be combined with current immunotherapies like checkpoint inhibitors or CAR-T cell therapy to synergistically impair tumor progression and extend patient survival.

## References

[B1] Al-Dalahmah O. Argenziano M. G. Kannan A. Mahajan A. Furnari J. Paryani F. (2023). Re-convolving the compositional landscape of primary and recurrent glioblastoma reveals prognostic and targetable tissue states. Nat. Commun. 14 (1), 2586. 10.1038/s41467-023-38186-1 37142563 PMC10160047

[B2] Anduran E. Dubois L. J. Lambin P. Winum J. Y. (2022). Hypoxia-activated prodrug derivatives of anti-cancer drugs: a patent review 2006 - 2021. Expert Opin. Ther. Pat. 32 (1), 1–12. 10.1080/13543776.2021.1954617 34241566

[B3] Bhaduri A. Di Lullo E. Jung D. Muller S. Crouch E. E. Espinosa C. S. (2020). Outer radial glia-like cancer stem cells contribute to heterogeneity of glioblastoma. Cell Stem Cell 26 (1), 48–63. 10.1016/j.stem.2019.11.015 31901251 PMC7029801

[B4] Brenner A. J. Floyd J. Fichtel L. Michalek J. Kanakia K. P. Huang S. (2021). Phase 2 trial of hypoxia activated evofosfamide (Th302) for treatment of recurrent bevacizumab-refractory glioblastoma. Sci. Rep. 11 (1), 2306. 10.1038/s41598-021-81841-0 33504881 PMC7841164

[B5] Chaligne R. Gaiti F. Silverbush D. Schiffman J. S. Weisman H. R. Kluegel L. (2021). Epigenetic encoding, heritability and plasticity of glioma transcriptional cell states. Nat. Genet. 53 (10), 1469–1479. 10.1038/s41588-021-00927-7 34594037 PMC8675181

[B6] Chanoch-Myers R. Wider A. Suva M. L. Tirosh I. (2022). Elucidating the diversity of malignant mesenchymal states in glioblastoma by integrative analysis. Genome Med. 14 (1), 106. 10.1186/s13073-022-01109-8 36123598 PMC9484143

[B7] Chen A. Liao S. Cheng M. Ma K. Wu L. Lai Y. (2022b). Spatiotemporal transcriptomic Atlas of mouse organogenesis using DNA nanoball-patterned arrays. Cell 185 (10), 1777–1792.e21. 10.1016/j.cell.2022.04.003 35512705

[B8] Chen X. Chen Y. Chen X. Wei P. Lin Y. Wu Z. (2022a). Single-cell rna sequencing reveals intra-tumoral heterogeneity of glioblastoma and a pro-tumor subset of tumor-associated macrophages characterized by Ezh2 overexpression. Biochim. Biophys. Acta Mol. Basis Dis. 1868 (12), 166534. 10.1016/j.bbadis.2022.166534 36057370

[B9] Cho C. S. Xi J. Si Y. Park S. R. Hsu J. E. Kim M. (2021). Microscopic examination of spatial transcriptome using seq-scope. Cell 184 (13), 3559–3572.e22. 10.1016/j.cell.2021.05.010 34115981 PMC8238917

[B10] Cho S. Y. Polster J. Engles J. M. Hilton J. Abraham E. H. Wahl R. L. (2006). *In vitro* evaluation of adenosine 5'-monophosphate as an imaging agent of tumor metabolism. J. Nucl. Med. 47 (5), 837–845. 16644754

[B11] Chryplewicz A. Scotton J. Tichet M. Zomer A. Shchors K. Joyce J. A. (2022). Cancer cell autophagy, reprogrammed macrophages, and remodeled vasculature in glioblastoma triggers tumor immunity. Cancer Cell 40 (10), 1111–1127.e9. 10.1016/j.ccell.2022.08.014 36113478 PMC9580613

[B12] Couturier C. P. Ayyadhury S. Le P. U. Nadaf J. Monlong J. Riva G. (2020). Single-cell rna-seq reveals that glioblastoma recapitulates a normal neurodevelopmental hierarchy. Nat. Commun. 11 (1), 3406. 10.1038/s41467-020-17186-5 32641768 PMC7343844

[B13] Coy S. Wang S. Stopka S. A. Lin J. R. Yapp C. Ritch C. C. (2022). Single cell spatial analysis reveals the topology of immunomodulatory purinergic signaling in glioblastoma. Nat. Commun. 13 (1), 4814. 10.1038/s41467-022-32430-w 35973991 PMC9381513

[B14] Dirkse A. Golebiewska A. Buder T. Nazarov P. V. Muller A. Poovathingal S. (2019). Stem cell-associated heterogeneity in glioblastoma results from intrinsic tumor plasticity shaped by the microenvironment. Nat. Commun. 10 (1), 1787. 10.1038/s41467-019-09853-z 30992437 PMC6467886

[B15] Dries R. Zhu Q. Dong R. Eng C. L. Li H. Liu K. (2021). Giotto: a toolbox for integrative analysis and visualization of spatial expression data. Genome Biol. 22 (1), 78. 10.1186/s13059-021-02286-2 33685491 PMC7938609

[B16] Duhamel M. Drelich L. Wisztorski M. Aboulouard S. Gimeno J. P. Ogrinc N. (2022). Spatial analysis of the glioblastoma proteome reveals specific molecular signatures and markers of survival. Nat. Commun. 13 (1), 6665. 10.1038/s41467-022-34208-6 36333286 PMC9636229

[B17] Eng C. L. Lawson M. Zhu Q. Dries R. Koulena N. Takei Y. (2019). Transcriptome-scale super-resolved imaging in tissues by rna seqfish. Nature 568 (7751), 235–239. 10.1038/s41586-019-1049-y 30911168 PMC6544023

[B18] Friebel E. Kapolou K. Unger S. Nunez N. G. Utz S. Rushing E. J. (2020). Single-cell mapping of human brain cancer reveals tumor-specific instruction of tissue-invading leukocytes. Cell 181 (7), 1626–1642. 10.1016/j.cell.2020.04.055 32470397

[B19] Fu W. Wang W. Li H. Jiao Y. Weng J. Huo R. (2020). Cytof analysis reveals a distinct immunosuppressive microenvironment in idh mutant anaplastic gliomas. Front. Oncol. 10, 560211. 10.3389/fonc.2020.560211 33614475 PMC7890006

[B20] Garofano L. Migliozzi S. Oh Y. T. D'Angelo F. Najac R. D. Ko A. (2021). Pathway-based classification of glioblastoma uncovers a mitochondrial subtype with therapeutic vulnerabilities. Nat. Cancer 2 (2), 141–156. 10.1038/s43018-020-00159-4 33681822 PMC7935068

[B21] Giesen C. Wang H. A. Schapiro D. Zivanovic N. Jacobs A. Hattendorf B. (2014). Highly multiplexed imaging of tumor tissues with subcellular resolution by mass cytometry. Nat. Methods 11 (4), 417–422. 10.1038/nmeth.2869 24584193

[B22] Goltsev Y. Samusik N. Kennedy-Darling J. Bhate S. Hale M. Vazquez G. (2018). Deep profiling of mouse splenic architecture with codex multiplexed imaging. Cell 174 (4), 968–981. 10.1016/j.cell.2018.07.010 30078711 PMC6086938

[B23] Goswami S. Raychaudhuri D. Singh P. Natarajan S. M. Chen Y. Poon C. (2023). Myeloid-specific Kdm6b inhibition sensitizes glioblastoma to Pd1 blockade. Nat. Cancer 4 (10), 1455–1473. 10.1038/s43018-023-00620-0 37653141

[B24] Guilhamon P. Chesnelong C. Kushida M. M. Nikolic A. Singhal D. MacLeod G. (2021). Single-cell chromatin accessibility profiling of glioblastoma identifies an invasive cancer stem cell population associated with lower survival. Elife 10, e64090. 10.7554/eLife.64090 33427645 PMC7847307

[B25] Hara T. Chanoch-Myers R. Mathewson N. D. Myskiw C. Atta L. Bussema L. (2021). Interactions between cancer cells and immune cells drive transitions to mesenchymal-like states in glioblastoma. Cancer Cell 39 (6), 779–792.e11. 10.1016/j.ccell.2021.05.002 34087162 PMC8366750

[B26] He S. Bhatt R. Brown C. Brown E. A. Buhr D. L. Chantranuvatana K. (2022). High-plex imaging of rna and proteins at subcellular resolution in fixed tissue by spatial molecular imaging. Nat. Biotechnol. 40 (12), 1794–1806. 10.1038/s41587-022-01483-z 36203011

[B27] Hegde A. Jayaprakash P. Couillault C. A. Piha-Paul S. Karp D. Rodon J. (2021). A phase I dose-escalation study to evaluate the safety and tolerability of evofosfamide in combination with ipilimumab in advanced solid malignancies. Clin. Cancer Res. 27 (11), 3050–3060. 10.1158/1078-0432.CCR-20-4118 33771853 PMC8172466

[B28] Ho P. C. Bihuniak J. D. Macintyre A. N. Staron M. Liu X. Amezquita R. (2015). Phosphoenolpyruvate is a metabolic checkpoint of anti-tumor T cell responses. Cell 162 (6), 1217–1228. 10.1016/j.cell.2015.08.012 26321681 PMC4567953

[B29] Hoogstrate Y. Draaisma K. Ghisai S. A. van Hijfte L. Barin N. de Heer I. (2023). Transcriptome analysis reveals tumor microenvironment changes in glioblastoma. Cancer Cell 41 (4), 678–692.e7. 10.1016/j.ccell.2023.02.019 36898379

[B30] Hua K. Li Y. Zhao Q. Fan L. Tan B. Gu J. (2018). Downregulation of annexin A11 (ANXA11) inhibits cell proliferation, invasion, and migration via the AKT/GSK-3β pathway in gastric cancer. Med. Sci. Monit. 24, 149–160. 10.12659/msm.905372 29306955 PMC5769363

[B31] Jain S. Rick J. W. Joshi R. S. Beniwal A. Spatz J. Gill S. (2023). Single-cell rna sequencing and spatial transcriptomics reveal cancer-associated fibroblasts in glioblastoma with protumoral effects. J. Clin. Invest. 133 (5), e147087. 10.1172/JCI147087 36856115 PMC9974099

[B32] Jayaprakash P. Ai M. Liu A. Budhani P. Bartkowiak T. Sheng J. (2018). Targeted hypoxia reduction restores T cell infiltration and sensitizes prostate cancer to immunotherapy. J. Clin. Invest. 128 (11), 5137–5149. 10.1172/JCI96268 30188869 PMC6205399

[B33] Johnson J. L. Dolezal M. C. Kerschen A. Matsunaga T. O. Unger E. C. (2009). *In vitro* comparison of dodecafluoropentane (ddfp), perfluorodecalin (pfd), and perfluoroctylbromide (pfob) in the facilitation of oxygen exchange. Artif. Cells Blood Substit. Immobil. Biotechnol. 37 (4), 156–162. 10.1080/10731190903043192 19548131

[B34] Johnson K. C. Anderson K. J. Courtois E. T. Gujar A. D. Barthel F. P. Varn F. S. (2021). Single-cell multimodal glioma analyses identify epigenetic regulators of cellular plasticity and environmental stress response. Nat. Genet. 53 (10), 1456–1468. 10.1038/s41588-021-00926-8 34594038 PMC8570135

[B35] Johnson Aljk J. Sall S. Laterra J. Lopez-Bertoni H. (2022). Stem-13 glioma stem cells mimic regulatory T cell function to suppress the immune response and promote tumor propagation. Neuro Oncol. 24, vii33–4. 10.1093/neuonc/noac209.130

[B36] Joseph J. V. Conroy S. Pavlov K. Sontakke P. Tomar T. Eggens-Meijer E. (2015). Hypoxia enhances migration and invasion in glioblastoma by promoting a mesenchymal shift mediated by the HIF1α-ZEB1 axis. Cancer Lett. 359 (1), 107–116. 10.1016/j.canlet.2015.01.010 25592037

[B37] Karimi E. Yu M. W. Maritan S. M. Perus L. J. M. Rezanejad M. Sorin M. (2023). Single-cell spatial immune landscapes of primary and metastatic brain tumours. Nature 614 (7948), 555–563. 10.1038/s41586-022-05680-3 36725935 PMC9931580

[B38] Kim Y. Danaher P. Cimino P. J. Hurth K. Warren S. Glod J. (2023). Highly multiplexed spatially resolved proteomic and transcriptional profiling of the glioblastoma microenvironment using archived formalin-fixed paraffin-embedded specimens. Mod. Pathol. 36 (1), 100034. 10.1016/j.modpat.2022.100034 36788070 PMC9937641

[B39] Kleino I. Frolovaite P. Suomi T. Elo L. L. (2022). Computational solutions for spatial transcriptomics. Comput. Struct. Biotechnol. J. 20, 4870–4884. 10.1016/j.csbj.2022.08.043 36147664 PMC9464853

[B40] Lee A. H. Sun L. Mochizuki A. Y. Reynoso J. G. Orpilla J. Chow F. (2021). Neoadjuvant Pd-1 blockade induces T cell and Cdc1 activation but fails to overcome the immunosuppressive tumor associated macrophages in recurrent glioblastoma. Nat. Commun. 12 (1), 6938. 10.1038/s41467-021-26940-2 34836966 PMC8626557

[B41] Lee J. H. Daugharthy E. R. Scheiman J. Kalhor R. Ferrante T. C. Terry R. (2015). Fluorescent *in situ* sequencing (fisseq) of rna for gene expression profiling in intact cells and tissues. Nat. Protoc. 10 (3), 442–458. 10.1038/nprot.2014.191 25675209 PMC4327781

[B42] Liau B. B. Sievers C. Donohue L. K. Gillespie S. M. Flavahan W. A. Miller T. E. (2017). Adaptive chromatin remodeling drives glioblastoma stem cell plasticity and drug tolerance. Cell Stem Cell 20 (2), 233–246. 10.1016/j.stem.2016.11.003 27989769 PMC5291795

[B43] Lickliter J. D. Ruben J. Kichenadasse G. Jennens R. Gzell C. Mason R. P. (2023). Dodecafluoropentane emulsion as a radiosensitizer in glioblastoma multiforme. Cancer Res. Commun. 3 (8), 1607–1614. 10.1158/2767-9764.CRC-22-0433 37609003 PMC10441549

[B44] Lin J. R. Fallahi-Sichani M. Sorger P. K. (2015). Highly multiplexed imaging of single cells using a high-throughput cyclic immunofluorescence method. Nat. Commun. 6, 8390. 10.1038/ncomms9390 26399630 PMC4587398

[B45] Liu S. Wang J. Guo C. Qi H. Sun M. Z. (2015). Annexin A11 knockdown inhibits *in vitro* proliferation and enhances survival of hca-F cell via akt2/foxo1 pathway and mmp-9 expression. Biomed. Pharmacother. 70, 58–63. 10.1016/j.biopha.2015.01.011 25776480

[B46] Liu Y. DiStasio M. Su G. Asashima H. Enninful A. Qin X. (2023). High-plex protein and whole transcriptome Co-mapping at cellular resolution with spatial cite-seq. Nat. Biotechnol. 41 (10), 1405–1409. 10.1038/s41587-023-01676-0 36823353 PMC10567548

[B47] Louis D. N. Perry A. Wesseling P. Brat D. J. Cree I. A. Figarella-Branger D. (2021). The 2021 who classification of tumors of the central nervous system: a summary. Neuro Oncol. 23 (8), 1231–1251. 10.1093/neuonc/noab106 34185076 PMC8328013

[B48] Loussouarn D. Oliver L. Salaud C. Samarut E. Bourgade R. Beroud C. (2023). Spatial distribution of immune cells in primary and recurrent glioblastoma: a small case study. Cancers (Basel) 15 (12), 3256. 10.3390/cancers15123256 37370866 PMC10297152

[B49] Ma T. Hu C. Lal B. Zhou W. Ma Y. Ying M. (2021). Reprogramming transcription factors Oct4 and Sox2 induce a brd-dependent immunosuppressive transcriptome in gbm-propagating cells. Cancer Res. 81 (9), 2457–2469. 10.1158/0008-5472.CAN-20-2489 33574085 PMC8137560

[B50] Ma Y. Zhou X. (2022). Spatially informed cell-type deconvolution for spatial transcriptomics. Nat. Biotechnol. 40 (9), 1349–1359. 10.1038/s41587-022-01273-7 35501392 PMC9464662

[B51] Medikonda R. Dunn G. Rahman M. Fecci P. Lim M. (2021). A review of glioblastoma immunotherapy. J. Neurooncol 151 (1), 41–53. 10.1007/s11060-020-03448-1 32253714

[B52] Merritt C. R. Ong G. T. Church S. E. Barker K. Danaher P. Geiss G. (2020). Multiplex digital spatial profiling of proteins and rna in fixed tissue. Nat. Biotechnol. 38 (5), 586–599. 10.1038/s41587-020-0472-9 32393914

[B53] Moffet J. J. D. Fatunla O. E. Freytag L. Kriel J. Jones J. J. Roberts-Thomson S. J. (2023). Spatial architecture of high-grade glioma reveals tumor heterogeneity within distinct domains. Neurooncol Adv. 5 (1), vdad142. 10.1093/noajnl/vdad142 38077210 PMC10699851

[B54] Murayama C. Kawaguchi A. T. Ishikawa K. Kamijo A. Kato N. Ohizumi Y. (2012). Liposome-encapsulated hemoglobin ameliorates tumor hypoxia and enhances radiation therapy to suppress tumor growth in mice. Artif. Organs 36 (2), 170–177. 10.1111/j.1525-1594.2011.01418.x 22339726

[B55] Neftel C. Laffy J. Filbin M. G. Hara T. Shore M. E. Rahme G. J. (2019). An integrative model of cellular states, plasticity, and genetics for glioblastoma. Cell 178 (4), 835–849. 10.1016/j.cell.2019.06.024 31327527 PMC6703186

[B56] Ostrom Q. T. Price M. Neff C. Cioffi G. Waite K. A. Kruchko C. (2023). Cbtrus statistical report: primary brain and other central nervous system tumors diagnosed in the United States in 2016-2020. Neuro Oncol. 25 (Suppl. ment_4), iv1–iv99. 10.1093/neuonc/noad149 37793125 PMC10550277

[B57] Patel A. P. Tirosh I. Trombetta J. J. Shalek A. K. Gillespie S. M. Wakimoto H. (2014). Single-cell rna-seq highlights intratumoral heterogeneity in primary glioblastoma. Science 344 (6190), 1396–1401. 10.1126/science.1254257 24925914 PMC4123637

[B58] Pham D. Tan X. Balderson B. Xu J. Grice L. F. Yoon S. (2023). Robust mapping of spatiotemporal trajectories and cell-cell interactions in healthy and diseased tissues. Nat. Commun. 14 (1), 7739. 10.1038/s41467-023-43120-6 38007580 PMC10676408

[B59] Przystal J. M. Becker H. Canjuga D. Tsiami F. Anderle N. Keller A. L. (2021). Targeting Csf1r alone or in combination with Pd1 in experimental glioma. Cancers (Basel) 13 (10), 2400. 10.3390/cancers13102400 34063518 PMC8156558

[B60] Puchalski R. B. Shah N. Miller J. Dalley R. Nomura S. R. Yoon J. G. (2018). An anatomic transcriptional Atlas of human glioblastoma. Science 360 (6389), 660–663. 10.1126/science.aaf2666 29748285 PMC6414061

[B61] Rashidi A. Billingham L. K. Zolp A. Chia T. Y. Silvers C. Katz J. L. (2024). Myeloid cell-derived creatine in the hypoxic niche promotes glioblastoma growth. Cell Metab. 36 (1), 62–77 e8. 10.1016/j.cmet.2023.11.013 38134929 PMC10842612

[B62] Ravi V. M. Neidert N. Will P. Joseph K. Maier J. P. Kuckelhaus J. (2022b). T-cell dysfunction in the glioblastoma microenvironment is mediated by myeloid cells releasing interleukin-10. Nat. Commun. 13 (1), 925. 10.1038/s41467-022-28523-1 35177622 PMC8854421

[B63] Ravi V. M. Will P. Kueckelhaus J. Sun N. Joseph K. Salie H. (2022a). Spatially resolved multi-omics deciphers bidirectional tumor-host interdependence in glioblastoma. Cancer Cell 40 (6), 639–655.e13. 10.1016/j.ccell.2022.05.009 35700707

[B64] Ren Y. Huang Z. Zhou L. Xiao P. Song J. He P. (2023). Spatial transcriptomics reveals niche-specific enrichment and vulnerabilities of radial glial stem-like cells in malignant gliomas. Nat. Commun. 14 (1), 1028. 10.1038/s41467-023-36707-6 36823172 PMC9950149

[B65] Richards L. M. Whitley O. K. N. MacLeod G. Cavalli F. M. G. Coutinho F. J. Jaramillo J. E. (2021). Gradient of developmental and injury response transcriptional states defines functional vulnerabilities underpinning glioblastoma heterogeneity. Nat. Cancer 2 (2), 157–173. 10.1038/s43018-020-00154-9 35122077

[B66] Sattiraju A. Kang S. Giotti B. Chen Z. Marallano V. J. Brusco C. (2023). Hypoxic niches attract and sequester tumor-associated macrophages and cytotoxic T cells and reprogram them for immunosuppression. Immunity 56 (8), 1825–1843.e6. 10.1016/j.immuni.2023.06.017 37451265 PMC10527169

[B67] Shekarian T. Zinner C. P. Bartoszek E. M. Duchemin W. Wachnowicz A. T. Hogan S. (2022). Immunotherapy of glioblastoma explants induces interferon-gamma responses and spatial immune cell rearrangements in tumor center, but not periphery. Sci. Adv. 8 (26), eabn9440. 10.1126/sciadv.abn9440 35776791 PMC10883360

[B68] Simonds E. F. Lu E. D. Badillo O. Karimi S. Liu E. V. Tamaki W. (2021). Deep immune profiling reveals targetable mechanisms of immune evasion in immune checkpoint inhibitor-refractory glioblastoma. J. Immunother. Cancer 9 (6), e002181. 10.1136/jitc-2020-002181 34083417 PMC8183210

[B69] Siska P. J. van der Windt G. J. Kishton R. J. Cohen S. Eisner W. MacIver N. J. (2016). Suppression of Glut1 and glucose metabolism by decreased akt/mtorc1 signaling drives T cell impairment in B cell leukemia. J. Immunol. 197 (6), 2532–2540. 10.4049/jimmunol.1502464 27511728 PMC5010978

[B70] Sottoriva A. Spiteri I. Piccirillo S. G. Touloumis A. Collins V. P. Marioni J. C. (2013). Intratumor heterogeneity in human glioblastoma reflects cancer evolutionary dynamics. Proc. Natl. Acad. Sci. U. S. A. 110 (10), 4009–4014. 10.1073/pnas.1219747110 23412337 PMC3593922

[B71] Sun R. Han R. McCornack C. Khan S. Tabor G. T. Chen Y. (2023). Trem2 inhibition triggers antitumor cell activity of myeloid cells in glioblastoma. Sci. Adv. 9 (19), eade3559. 10.1126/sciadv.ade3559 37172094 PMC10181199

[B72] Taylor M. J. Lukowski J. K. Anderton C. R. (2021). Spatially resolved mass spectrometry at the single cell: recent innovations in proteomics and metabolomics. J. Am. Soc. Mass Spectrom. 32 (4), 872–894. 10.1021/jasms.0c00439 33656885 PMC8033567

[B73] Teicher B. A. Ara G. Herbst R. Takeuchi H. Keyes S. Northey D. (1997). Peg-hemoglobin: effects on tumor oxygenation and response to chemotherapy. Vivo 11 (4), 301–311. 9292296

[B74] van Hooren L. Handgraaf S. M. Kloosterman D. J. Karimi E. van Mil L. Gassama A. A. (2023). Cd103(+) regulatory T cells underlie resistance to radio-immunotherapy and impair Cd8(+) T cell activation in glioblastoma. Nat. Cancer 4 (5), 665–681. 10.1038/s43018-023-00547-6 37081259 PMC10212765

[B75] Varn F. S. Johnson K. C. Martinek J. Huse J. T. Nasrallah M. P. Wesseling P. (2022). Glioma progression is shaped by genetic evolution and microenvironment interactions. Cell 185 (12), 2184–2199.e16. 10.1016/j.cell.2022.04.038 35649412 PMC9189056

[B76] Verhaak R. G. Hoadley K. A. Purdom E. Wang V. Qi Y. Wilkerson M. D. (2010). Integrated genomic analysis identifies clinically relevant subtypes of glioblastoma characterized by abnormalities in pdgfra, Idh1, egfr, and Nf1. Cancer Cell 17 (1), 98–110. 10.1016/j.ccr.2009.12.020 20129251 PMC2818769

[B77] Vickovic S. Eraslan G. Salmen F. Klughammer J. Stenbeck L. Schapiro D. (2019). High-definition spatial transcriptomics for *in situ* tissue profiling. Nat. Methods 16 (10), 987–990. 10.1038/s41592-019-0548-y 31501547 PMC6765407

[B78] Wang E. J. Chen J. S. Jain S. Morshed R. A. Haddad A. F. Gill S. (2021). Immunotherapy resistance in glioblastoma. Front. Genet. 12, 750675. 10.3389/fgene.2021.750675 34976006 PMC8718605

[B79] Wang L. Jung J. Babikir H. Shamardani K. Jain S. Feng X. (2022). A single-cell Atlas of glioblastoma evolution under therapy reveals cell-intrinsic and cell-extrinsic therapeutic targets. Nat. Cancer 3 (12), 1534–1552. 10.1038/s43018-022-00475-x 36539501 PMC9767870

[B80] Wang Q. Hu B. Hu X. Kim H. Squatrito M. Scarpace L. (2017). Tumor evolution of glioma-intrinsic gene expression subtypes associates with immunological changes in the microenvironment. Cancer Cell 32 (1), 42–56. 10.1016/j.ccell.2017.06.003 28697342 PMC5599156

[B81] Wang X. Allen W. E. Wright M. A. Sylwestrak E. L. Samusik N. Vesuna S. (2018). Three-dimensional intact-tissue sequencing of single-cell transcriptional states. Science 361 (6400), eaat5691. 10.1126/science.aat5691 29930089 PMC6339868

[B82] Wang Y. Liu B. Zhao G. Lee Y. Buzdin A. Mu X. (2023a). Spatial transcriptomics: technologies, applications and experimental considerations. Genomics 115 (5), 110671. 10.1016/j.ygeno.2023.110671 37353093 PMC10571167

[B83] Wang Z. Zhu M. Dong R. Cao D. Li Y. Chen Z. (2023b). Th-302-Loaded nanodrug reshapes the hypoxic tumour microenvironment and enhances Pd-1 blockade efficacy in gastric cancer. J. Nanobiotechnology 21 (1), 440. 10.1186/s12951-023-02203-8 37993847 PMC10664313

[B84] Wen P. Y. Weller M. Lee E. Q. Alexander B. M. Barnholtz-Sloan J. S. Barthel F. P. (2020). Glioblastoma in adults: a society for neuro-oncology (sno) and European society of neuro-oncology (eano) consensus review on current management and future directions. Neuro Oncol. 22 (8), 1073–1113. 10.1093/neuonc/noaa106 32328653 PMC7594557

[B85] Xiao Y. Wang Z. Zhao M. Deng Y. Yang M. Su G. (2022). Single-cell transcriptomics revealed subtype-specific tumor immune microenvironments in human glioblastomas. Front. Immunol. 13, 914236. 10.3389/fimmu.2022.914236 35669791 PMC9163377

[B86] Xiong A. Zhang J. Chen Y. Zhang Y. Yang F. (2022). Integrated single-cell transcriptomic analyses reveal that gpnmb-high macrophages promote pn-mes transition and impede T cell activation in gbm. EBioMedicine 83, 104239. 10.1016/j.ebiom.2022.104239 36054938 PMC9437813

[B87] Yabo Y. A. Niclou S. P. Golebiewska A. (2022). Cancer cell heterogeneity and plasticity: a paradigm shift in glioblastoma. Neuro Oncol. 24 (5), 669–682. 10.1093/neuonc/noab269 34932099 PMC9071273

[B88] Ye X. Z. Xu S. L. Xin Y. H. Yu S. C. Ping Y. F. Chen L. (2012). Tumor-associated microglia/macrophages enhance the invasion of glioma stem-like cells via TGF-β1 signaling pathway. J. Immunol. 189 (1), 444–453. 10.4049/jimmunol.1103248 22664874

[B89] Yuan J. Levitin H. M. Frattini V. Bush E. C. Boyett D. M. Samanamud J. (2018). Single-cell transcriptome analysis of lineage diversity in high-grade glioma. Genome Med. 10 (1), 57. 10.1186/s13073-018-0567-9 30041684 PMC6058390

[B90] Yuan Y. Bar-Joseph Z. (2020). Gcng: graph convolutional networks for inferring gene interaction from spatial transcriptomics data. Genome Biol. 21 (1), 300. 10.1186/s13059-020-02214-w 33303016 PMC7726911

[B91] Zhang M. Hutter G. Kahn S. A. Azad T. D. Gholamin S. Xu C. Y. (2016). Anti-Cd47 treatment stimulates phagocytosis of glioblastoma by M1 and M2 polarized macrophages and promotes M1 polarized macrophages *in vivo* . PLoS One 11 (4), e0153550. 10.1371/journal.pone.0153550 27092773 PMC4836698

[B92] Zhang P. Rashidi A. Zhao J. Silvers C. Wang H. Castro B. (2023). Sting agonist-loaded, Cd47/Pd-L1-Targeting nanoparticles potentiate antitumor immunity and radiotherapy for glioblastoma. Nat. Commun. 14 (1), 1610. 10.1038/s41467-023-37328-9 36959214 PMC10036562

[B93] Zheng Y. Carrillo-Perez F. Pizurica M. Heiland D. H. Gevaert O. (2023). Spatial cellular architecture predicts prognosis in glioblastoma. Nat. Commun. 14 (1), 4122. 10.1038/s41467-023-39933-0 37433817 PMC10336135

[B94] Zhou R. Yang G. Zhang Y. Wang Y. (2023). Spatial transcriptomics in development and disease. Mol. Biomed. 4 (1), 32. 10.1186/s43556-023-00144-0 37806992 PMC10560656

